# A phospholipid transfer function of ER-mitochondria encounter structure revealed in vitro

**DOI:** 10.1038/srep30777

**Published:** 2016-07-29

**Authors:** Rieko Kojima, Toshiya Endo, Yasushi Tamura

**Affiliations:** 1 Department of Material and Biological Chemistry, Faculty of Science, Yamagata University, 1-4-12, Kojirakawa-machi, Yamagata, Yamagata 990-8560, Japan; 2 Faculty of Life Sciences, Kyoto Sangyo University, Kamigamo-motoyama, Kita-ku, Kyoto 603-8555, Japan

## Abstract

As phospholipids are synthesized mainly in the endoplasmic reticulum (ER) and mitochondrial inner membranes, how cells properly distribute specific phospholipids to diverse cellular membranes is a crucial problem for maintenance of organelle-specific phospholipid compositions. Although the ER-mitochondria encounter structure (ERMES) was proposed to facilitate phospholipid transfer between the ER and mitochondria, such a role of ERMES is still controversial and awaits experimental demonstration. Here we developed a novel *in vitro* assay system with isolated yeast membrane fractions to monitor phospholipid exchange between the ER and mitochondria. With this system, we found that phospholipid transport between the ER and mitochondria relies on membrane intactness, but not energy sources such as ATP, GTP or the membrane potential across the mitochondrial inner membrane. We further found that lack of the ERMES component impairs the phosphatidylserine transport from the ER to mitochondria, but not the phosphatidylethanolamine transport from mitochondria to the ER. This *in vitro* assay system thus offers a powerful tool to analyze the non-vesicular phospholipid transport between the ER and mitochondria.

In eukaryotic cells, complex membrane structures called organelles are developed and exert distinct functions to perform various cellular activities. The organelle functions strictly rely on their resident proteins and properly maintained lipid compositions. Organellar proteins are mostly synthesized in the cytosol and transported to specific organelles, and knowledge has been extensively accumulated on the pathways and factors for protein transport to the endoplasmic reticulum (ER), mitochondria, chloroplasts, and peroxisomes. On the other hand, little is known about pathways, factors, and molecular mechanisms of phospholipid trafficking among different organellar membranes^1^,^2^,^3^,^4^.

In yeast, two abundant phospholipids, phosphatidylcholine (PC) and phosphatidylethanolamine (PE), are mainly synthesized from phosphatidylserine (PS), which is generated in the ER^5^,^6^ (Fig. 1A). Newly synthesized PS is transported from the ER to mitochondria and subsequently decarboxylated to become PE by Psd1, which is located in the mitochondrial inner membrane (IM)^7^,^8^ (Fig. 1A). A part of PE is transported back across the mitochondrial outer membrane (OM) to the ER and then converted to PC through methylation by the ER-resident enzymes, Cho2 and Opi3^9^ (Fig. 1A). Syntheses of PE and PC thus strongly depend on inter-organelle transport of their precursor phospholipids.

Phospholipid transport across an aqueous phase between the organellar membranes that are not connected by vesicular transport is enigmatic. Since phospholipid molecules with two hydrophobic acyl chains spontaneously exchange between the membranes only very slowly, specific mechanisms to facilitate such a process should be operated in the cell^4^. For example, soluble lipid transfer proteins can shuttle between the membranes to promote the exchange of phospholipids between the membranes. Ups1-Mdm35 and Ups2-Mdm35, evolutionary conserved mitochondrial protein complexes in the mitochondrial intermembrane space (IMS) were found to mediate phosphatidic acid (PA) and PS transfers, respectively, between the mitochondrial OM and IM^10^,^11^,^12^,^13^,^14^,^15^. Ups3, an two evolutionary conserved Ups1 homologous protein in the IMS, also form a complex with Mdm35, and may participate in phospholipid transport in the IMS although its possible substrate phospholipids have not been identified^16^,^17^,^18^,^19^,^20^.

Alternatively, close membrane contacts could facilitate the spontaneous transfer or protein-mediated transfer of phospholipids between the membranes^21^. Recent identification of membrane tethering proteins for different organellar membranes supports this idea. A molecular tether between the ER and OM termed the ERMES (ER-Mitochondria Encounter Structure) was identified and proposed to facilitate phospholipid exchange between the ER and mitochondria^22^. ERMES consists of four core subunits, Mmm1, Mdm34, Mdm12, and Mdm10, and two peripheral proteins, Gem1 and Tom7^22^,^23^,^24^. Among them, Mmm1, Mdm34 and Mdm12 contain a SMP (synaptotagmin-like, mitochondrial and lipid-binding proteins) domain, which could bind to hydrophobic substrates for transport. Indeed, Mmm1 and Mdm12 were shown to bind phospholipids^25^,^26^. However, possible phospholipid transfer by ERMES is still controversial because *in vivo* and *in vitro* analyses showed that loss of an ERMES subunit leads to only minor defects in phospholipid transport from the ER to mitochondria as measured by the PS to PE conversion^27^,^28^. A similar contact between the ER and OM termed EMC (a conserved endoplasmic reticulum membrane protein complex) was identified and considered to facilitate phospholipid transfer from the ER to mitochondria, as well^29^. In addition to the ER-mitochondria contacts, a new contact was identified between mitochondria and the vacuole, which is connected to the ER by vesicular transport, and named vCLAMP (vacuole and mitochondria patch). vCLAMP was also suggested to facilitate the phospholipid exchange between the OM and vacuolar membrane, yet its role becomes prominent when ERMES functions are defective^30^,^31^. Lam6/Ltc1 is localized to both ER-mitochondria and ER-vacuole contact regions and likely involved in transfer of sterols between membranes^32^,^33^. Interestingly, loss of ERMES expands the area of vCLAMP, and in turn, loss of vCLAMP components increases the ER-mitochondrial contacts consisting of ERMES^32^. This indicates that phospholipid transport pathways are redundant and one of the pathways involving mitochondria can be bypassed by changing the lipid transport pathways via distinct mitochondrial contacts with other organelles. Supporting this idea, a recent study demonstrates that expression of dominant mutants of an endosomal protein Vps13 compensates loss of ERMES functions^34^. Such an inherently robust nature of phospholipid trafficking naturally makes it difficult to test the direct role and contribution of each phospholipid transfer protein or pathway in intracellular phospholipid transport *in vivo.*

To assess the mechanisms of phospholipid transport, an *in vitro* phospholipid transport assay using isolated cellular membranes could provide a powerful and straightforward means^35^. However, currently available *in vitro* phospholipid transport assay systems are mainly designed to analyze the PS import from the ER into mitochondria, but not the PE export from mitochondria to the ER^7^,^27^,^28^,^36^. In principle, export of PE, which is converted from PS at the IM, from mitochondria to the ER can be monitored by measuring the radioactive PE to PC conversions. However, efficiency of PC production is generally much lower than those of PS and PE, suggesting that the PE export or PC synthesis remains ineffective *in vitro*^37^,^38^. We thus decided to develop a novel *in vitro* assay system so that we could analyze not only PS import into mitochondria from the ER but also PE export from mitochondria to the ER. The *in vitro* assay system is free from secondary effects owing to the presence of other organelle membranes such as endosomes or the vacuole, which could also be the sources of phospholipid supply *in vivo*. By using this *in vitro* assay system, we examined the fundamental properties of mitochondria-ER phospholipid transport, such as energy requirements, like the needs of the membrane potential across the IM (∆Ψ) and/or hydrolysis of nucleotide triphosphates (ATP and GTP). Besides importantly, we could show that ERMES components are involved in phospholipid transport between mitochondria and the ER.

## Results

### Heavy membrane fraction with high phospholipid synthetic activities

**Figure 1:**
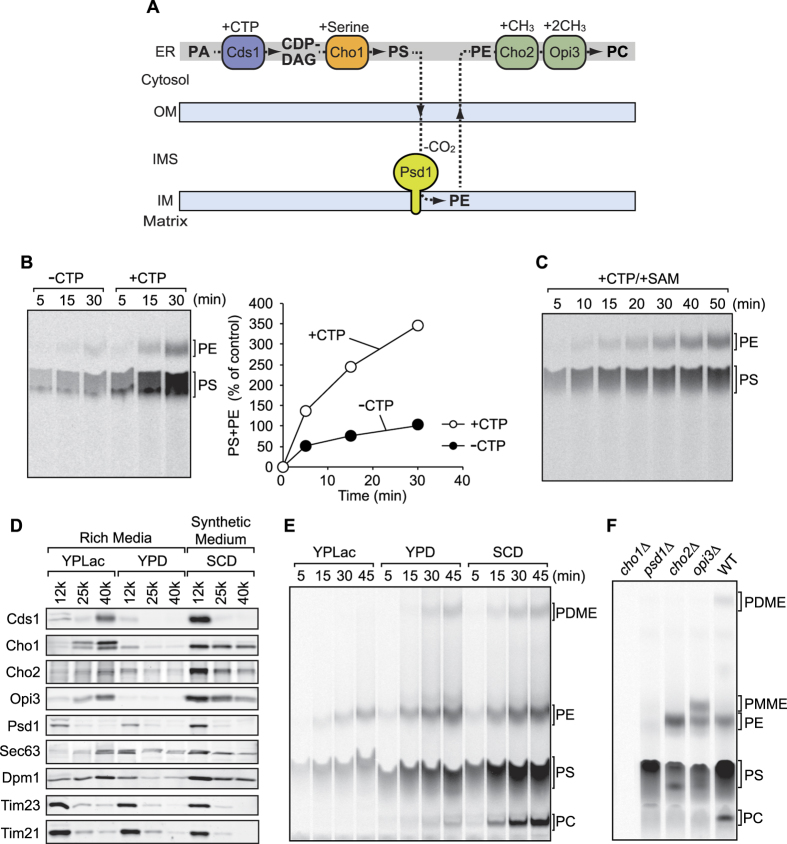
Fermentable and low-nutrient conditions are suitable for isolation of membrane fractions containing both the ER and mitochondria. (**A**) Phospholipid biosynthetic pathways in the ER and mitochondria. PA, phosphatidic acid; CDP-DAG, CDP-diacylglycerol; PS, phosphatidylserine; PE, phosphatidylethanolamine; PC, phosphatidylcholine; OM, mitochondrial outer membrane; IMS, intermembrane space; IM, mitochondrial inner membrane. (**B**) The heavy membrane fractions were incubated with [^14^C]-serine in the presence or absence of 2 mM CTP at 30 °C for the indicated time. Total phospholipids were extracted and analyzed by TLC and radioimaging. The total amount of synthesized phospholipid (PS+PE) in the absence of CTP after 30 min incubation was set to 100%. (**C**) PS was synthesized *in vitro* using the same membrane fractions as in (**B**) in the presence of CTP and S-adenosylmethionine. (**D**) Yeast cells cultivated in YPLac, YPD or SCD were subjected to subcellular fractionation. 12 k, 25 k and 40 k pellets were analyzed by SDS-PAGE followed by immunoblotting using the indicated antibodies against phospholipid synthetic enzymes, ER and mitochondrial marker proteins. (**E**) The 12 k pellet fractions isolated from yeast cells cultivated in YPLac, YPD or SCD were incubated with [^14^C]-serine for the indicated times. Total phospholipids were extracted and analyzed by TLC and radioimaging. (**F**) 12 k pellet prepared from wild-type cells or mutant cells lacking a phospholipid synthetic enzyme were incubated with [^14^C]-serine and synthesized radiolabeled phospholipids were analyzed as in (**B**).

In the conventional *in vitro* phospholipid transport assay, one can follow the fate of radioactive PS, which can be generated by incubation of radioactive [^14^C]-serine with the isolated yeast membrane fraction containing mitochondria and the ER membrane. This assay system thus relies on a coupling reaction of the high-energy intermediate CDP-diacylglycerol (CDP-DAG) with radioactive serine to form radioactive PS, which is catalyzed by Cho1 in the ER membrane (Fig. 1A). Then PS can be transported from the ER to mitochondria to be converted to PE by Psd1 in the IM (Fig. 1A). Therefore one can expect that an increase in the CDP-DAG level may lead to enhanced PS synthesis and subsequently enhanced PE synthesis. To test this idea, we isolated a heavy membrane fraction (HMF, 12,000× *g* pellet, see materials and methods) from yeast cells that had been cultivated in a non-fermentable medium, YPLac. Then we incubated the HMF with [^14^C]-serine in the presence or absence of CTP *in vitro*. Time-dependent increases in the levels of PS and then of PE (Fig. 1B) reflect the capability of the HMF to produce PS and PE, indicating that it contains both the ER and mitochondrial membranes and that newly synthesized PS is transported from the ER to mitochondria. Importantly, PS was efficiently synthesized only when we added CTP to the reaction (Fig. 1B). Therefore we can monitor the PS import from the ER into mitochondria efficiently in the presence of CTP, but not upon omission of CTP, *in vitro.* PE can be transported from mitochondria to the ER, where Cho2 and Opi3 mediate conversion of PE to PC by using a methyl source, S-adenosylmethionine (SAM) (Fig. 1A). However, we could not detect PC production at all even in the presence of SAM despite the efficient synthesis of PS and its subsequent conversion to PE in a time-dependent manner (Fig. 1C). Apparently PE export from the mitochondria to the ER is inefficient *in vitro* or the HMF does not contain active Cho2 or Opi3.

To find out the reason for the low efficiency of the PC synthesis in the *in vitro* phospholipid transport assays, we analyzed the levels of phospholipid synthetic enzymes in the heavy (12,000 × *g* pellet) as well as light membrane fractions (LMFs, 25,000 and 40,000 × *g* pellets) prepared from yeast cells cultivated in YPLac medium. Immunoblotting showed that ER-resident phospholipid synthetic enzymes such as Cds1, Cho1, Cho2 and Opi3 were enriched in the LMFs like ER-marker proteins Sec63 and Dpm1 (Fig. 1D, YPLac, 25 k and 40 k) while mitochondrial proteins (Psd1, Tim23 and Tim21) were mainly present in the HMF (Fig. 1D, YPLac, 12 k). These results explain well why the HMF is not able to produce PC (Fig. 1C) although the residual level of the ER resident PS synthetic enzyme, Cho1, appeared sufficient to synthesize PS (Fig. 1B,C).

Since the levels and distribution between the HMF and LMF of phospholipid synthetic enzymes may be affected by culturing conditions, we cultivated yeast cells in fermentable media (YPD and SCD) and isolated the HMF and LMF. Interestingly, both mitochondria- and ER-resident proteins were co-fractionated in the HMFs when cells were cultivated in fermentable media although it is obvious that intact ER structure is disrupted during the membrane preparation (Fig. 1D, YPD and SCD, 12k). These results suggest that the density of the ER or the ER-mitochondria tethering tends to increase under fermentable conditions. We also note that the steady state levels of phospholipid synthetic enzymes dramatically increase when cells are grown in the synthetic medium, SCD, as compared with YPD. The increased levels of phospholipid synthetic enzymes are probably due to a lower concentration of inositol in SCD, which is a negative regulator for transcriptions of genes encoding phospholipid synthetic enzymes^5^.

Consistent with these observations, phospholipid synthetic activities *in vitro* were significantly improved when the HMF isolated from the SCD-grown cells were used instead of those from the YPLac-grown cells (Fig. 1E). Moreover, the HMF prepared from the SCD-grown cells generated PC efficiently via the intermediate phosphatidyldimethylethanolamine (PDME) *in vitro* (Fig. 1E, SCD). We further tested the *in vitro* phospholipid syntheses by using the HMF prepared from the SCD-grown cells that lack phospholipid synthetic enzymes. Upon incubation with radiolabeled serine, the HMF lacking Cho1 failed to generate PS and its downstream phospholipid products like PE and PC, as expected (Fig. 1F). The HMF that lacks Psd1, Cho2 or Opi3 did not produce PC as well, but instead accumulated precursor phospholipids such as PS (*psd1*∆), PE (*cho2*∆) or phosphatidylmonomethylethanolamine (PMME) (*opi3*∆) (Fig. 1F). Since no PE was generated in the absence of Psd1 (*psd1*Δ), endosome-resident enzyme Psd2^39^ was not involved in this assay system (Fig. 1F). These results indicate that the present *in vitro* assay system allows us to monitor the synthesis and transport of phospholipids involving the ER and mitochondria, but not endosomes or the vacuole.

### Optimization of *in vitro* assay conditions

We further optimized the conditions for the *in vitro* PS synthesis and transport; we determined the proper pH and concentrations of Mn^2+^, which was reported to increase the enzymatic activity of Cho1^40^, CTP, and SAM to monitor phospholipid transport between the ER and mitochondria (Fig. S1). We have now established the optimized *in vitro* assay system by using the isolated HMF to follow the synthesis of PS, PDME, and PC in the ER and of PE in mitochondria. To characterize this system further, we tested temperature dependence of the phospholipid synthesis with this system (Fig. S2). When we incubated the HMFs with [^14^C]-serine on ice, little PS was produced and almost no PE or PC was detected even after 45 min of incubation (Fig. S2). When we increased temperature to 16 °C, we still observe only small amounts of PS, PE and PC generated as compared with those at 30 °C. Therefore our *in vitro* assay system requires physiological temperature for PS synthesis and transport.

We next asked whether our *in vitro* assay system requires intact organelle membranes for efficient PS synthesis and transport. We thus pretreated the isolated HMFs with low concentrations of Triton X-100, and then added [^14^C]-serine to initiate the PS synthesis. Although PS was normally synthesized after solubilization of the HMFs, conversion of PS to PE or PC was nearly abolished by the detergent solubilization of the membranes (Fig. S2). Therefore at least PE synthesis requires intact organelle membranes.

### Energy requirements

**Figure 2:**
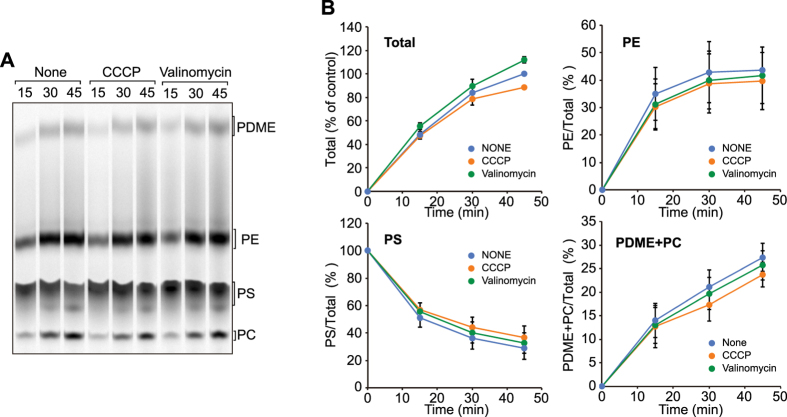
∆Ψ is dispensable for *in vitro* PS synthesis and transport. (**A**) *In vitro* PS transport assays were performed using the heavy membrane fractions pretreated with 60 μM CCCP or 20 μg/ml valinomycin. (**B**) Amounts of PS, PE and PDME+PC relative to total phospholipids were calculated and plotted. Values are mean ± SEM (*n* = 3). The amount of total phospholipids synthesized with the wild-type membranes after 45 min incubation was set to 100% (Total).

A previous study using unilamellar phospholipid liposomes containing [^3^H]-labeled PS and isolated mitochondria showed that import of radioactive PS from liposomes to mitochondria, as monitored by conversion of PS to PE, does not require the membrane potential across the IM (∆Ψ)^7^. We thus asked if PS import from the ER into mitochondria and PE export from mitochondria to the ER require the ∆Ψ in the present *in vitro* assay system. We pretreated the HMFs with a protonophore, CCCP (carbonyl cyanide m-chlorophenylhydrazone), or K^+^-specific ionophore, valinomycin, to dissipate ∆Ψ, and added [^14^C]-serine to initiate the PS synthesis and subsequent transport. The time course of the synthesis of radioactive PE and PC showed that they were not affected by the loss of ∆Ψ, eliminating the active roles of ΔΨ in the phospholipid synthetic reactions and transport processes of PS and PE (Fig. 2).

**Figure 3:**
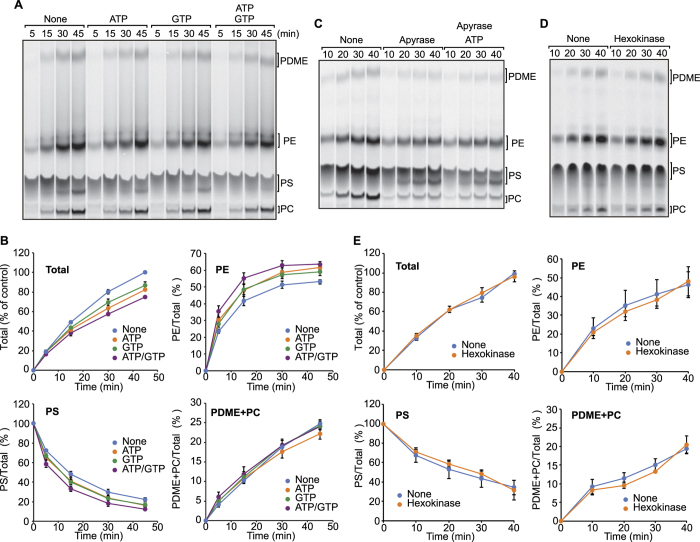
ATP and GTP supplements do not drastically affect PS synthesis or transport *in vitro*. (**A**) *In vitro* PS transport assays were performed in the absence (None) or presence of 2 mM ATP, 2 mM GTP or 2 mM ATP and GTP. (**B**) Quantifications of total phospholipids (Total) and relative amounts of PS, PE and PDME+PC relative to total phospholipids synthesized in (**A**). Total phospholipids synthesized with the wild-type membranes after 45 min incubation was set to 100%. Values are mean ± SEM (*n* = 3). (**C**) Heavy membrane fractions pretreatment with boiled apyrase (None) or apyrase (Apyrase) were used to analyze PS transport in the presence or absence of ATP. (**D**) Heavy membrane fractions were pretreated with hexokinase (Hexokinase) or buffer (None) in the presence of glucose to deplete ATP were subjected to the *in vitro* PS transport assay. (**E**) Quantifications of phospholipids synthesized in (**D**) were performed as in (**B**).

Previous studies using intact or permeabilized mammalian cells showed that the PS import from the ER into mitochondria is ATP dependent^41^,^42^,^43^,^44^. However other studies using isolated organelles indicated that the PS import does not require ATP^7^,^45^. We thus re-examined the effects of nucleotide triphosphates such as ATP and GTP on the phospholipid transport in the present *in vitro* assay system (Fig. 3). Although addition of ATP and/or GTP slightly declined the PS synthesis and led to slight increases in the PS to PE conversion, it did not affect generations of PC and PDME at all (Fig 3A,B). Therefore, at least in the present *in vitro* assay system, neither ATP nor GTP has stimulatory effects on the phospholipid transport between the ER and mitochondria.

To further confirm that ATP is not required for the phospholipid transport between the ER and mitochondria *in vitro*, we pretreated the HMFs with potato apyrase to deplete ATP and subjected them to the PS transport analyses *in vitro*. Unexpectedly, apyrase treatments inhibited both PS synthesis and PS conversions to PE and PC (Fig. 3C, Apyrase). However, re-addition of ATP after ATP depletion by apyrase did not restore the defective PS synthesis and its conversion to PE and PC (Fig. 3C, Apyrase ATP). Therefore apparent inhibition of PS synthesis and its conversion to PE and PC are likely due to unknown side effects of the apyrase treatment. Indeed, when we used hexokinase instead of apyrase for depletion of ATP, we observed no effect on PS synthesis or its conversion to PE and PC (Fig. 3D,E). Taken these results together, we conclude that ATP is not required for the PS or PE transport, meaning that these transport processes proceed by facilitated passive diffusion.

### ERMES plays an important role in phospholipid transport

The present optimized *in vitro* assay system by using the isolated HMF allows us to monitor not only the PS transport, as reflected in the relative amount of PS to radioactive total phospholipids (PS/Total), but also the PE transport, as reflected in the relative amount of radioactive PDME+PC to radioactive PE ((PDME+PC)/PE). When we increased the level of Psd1 in the cell, which converts PS to PE, the rate of PE/Total did not change significantly (Fig. S3), suggesting that Psd1 molecules were not saturated by imported PS molecules under the present conditions. Similarly, an increase in the level of Cho2 or Opi3, which catalyzes methylations of PE or PMME and PDME, respectively, did not affect the rate of (PDME+PC)/PE significantly (Fig. S3). Therefore in the present *in vitro* assay system, transport processes of PS and PE, not their corresponding enzymatic reactions, limit the apparent rates of changes in the amounts of PS, PE and PDME+PC.

By using this system, we asked if loss of the component(s) of ERMES would affect PS and PE transport processes *in vitro* since involvement of ERMES in the ER-mitochondria phospholipid exchange is still in debate. Originally, Kornmann *et al.* showed that loss of Mmm1, Mdm34, Mdm10 or Mdm12 decelerates the PS to PC conversion^22^. However in the later *in vivo* and *in vitro* studies, two groups showed that loss of an ERMES subunit does not cause a defect in the PS to PE conversion^27^,^28^. To minimize the potential secondary effects arising from the lack of ERMES component(s), we prepared the HMFs from wild-type, *mmm1∆* and *mdm12∆* cells expressing Vps13-D716H. Vps13-D716H is a “dominant positive” mutant of an endosomal protein Vps13 and its expression rescues the growth defects in ERMES-lacking cells^34^.

**Figure 4:**
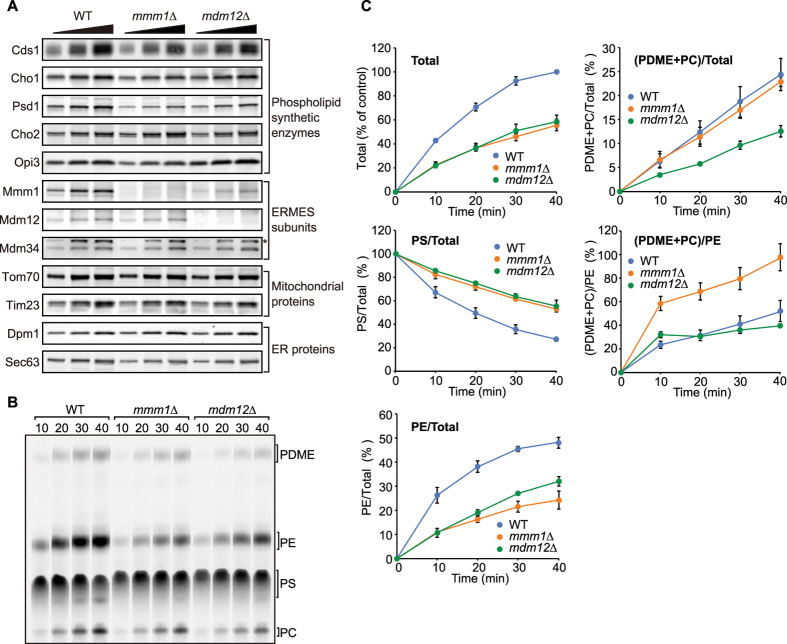
ERMES facilitates phospholipid transport between the ER and mitochondria. (**A**) Proteins in the heavy membrane fractions isolated from wild-type, *mmm1∆* and *mdm12∆* cells expressing Vps13-D716H were analyzed by SDS-PAGE followed by immunoblotting using the indicated antibodies. The asterisk indicates a nonspecific band. (**B**) *In vitro* PS transport assays were performed using the heavy membrane fractions isolated from wild-type, *mmm1∆* and *mdm12∆* cells expressing Vps13-D716H. (**C**) Amounts of PS, PE and PDME+PC relative to total phospholipids were calculated and plotted. Values are mean ± SEM (*n* = 4). The amount of total phospholipids synthesized with wild-type cells after 40 min incubation was set to 100% (Total).

We first analyzed the steady-state levels of phospholipid synthetic enzymes contained in the prepared HMF by immunoblotting. As shown in Fig. 4A, comparable amounts of the PS synthase, Cho1, and PE methyltransferases, Cho2 and Opi3, were present in the wild-type, *mmm1*∆ and *mdm12*∆ HMFs, yet the levels of CDS-DAG synthase, Cds1, and PE synthase, Psd1, were slightly lower in the *mmm1∆* and *mdm12*∆ HMFs than the wild-type HMF (Fig. 4A). Mmm1 was destabilized in the absence of Mdm12 while loss of Mmm1 did not significantly affect stabilities of the other ERMES subunits, Mdm12 and Mdm34 (Fig. 4A).

We incubated the HMFs with [^14^C]-serine for different periods of time and analyzed synthesized phospholipids by TLC followed by radioimaging (Fig. 4B). Radioactive phospholipids were quantified and the amounts of each phospholipid to total radioactive phospholipids or PE were plotted against incubation time (Fig. 4C). The relative amounts of PS to total phospholipids (PS/Total) efficiently decreased in a time-dependent manner in wild-type HMF while PS synthesis, which was reflected in the total phospholipids (Total), lasted for as long as 40 min of incubation (Fig. 4C, WT, PS/Total and Total). This decrease in PS/Total reflects the PS transport from the ER to mitochondria. Strikingly, the loss of Mmm1 or Mdm12 decreased the PS transport rates as compared with that of the wild-type HMF (Fig. 4B,C). Consistently, the increase in the amounts of PE relative to total phospholipids (PE/Total) became slower in the absence of Mmm1 or Mdm12 than the wild-type HMF (Fig. 4B,C). Essentially the same results were obtained when we used *mdm34∆* HMFs (Fig. S4). We also observed the slowed PS transport in the absence of Mmm1 when Psd1 is overexpressed, indicating that the compromised PS transport and PE synthesis are not due to the decreased Psd1 levels in the absence of Mmm1 (Fig. S5). Of note, the PS synthesis on its own decreased by ~50% in *mmm1*∆ and *mdm12*∆ HMFs as compared with that in the wild-type HMF (Fig. 4B,C, Total). However, when we synthesized reduced amounts of PS *in vitro* using the half amounts of [^14^C]-serine, the ratios of PS, PE and PDME+PC to total radioactive phospholipids were not altered, demonstrating that the decreased PS synthesis is not the reason for the delayed rate of the PS transport (Fig. S6). Taken together, we conclude that ERMES plays a critical role in facilitating the PS transport from the ER to mitochondria.

In contrast to the PS transport from the ER to mitochondria, the PE transport from mitochondria to the ER, which can be reflected in the relative amounts of PDME+PC to PE ((PDME+PC)/PE), appeared less dependent on the ERMES complex. The PE to PDME/PC conversion rates were similar between wild-type and *mdm12*∆ HMFs while it was even accelerated in *mmm1*∆ and *mdm34∆* HMFs (Figs 4 and S4, (PDME+PC)/PE). The *mmm1∆mdm12∆* HMFs showed essentially the similar results to the *mmm1*∆ HMFs, meaning that *MMM1* is epistatic to *MDM12* (Fig. S7B,C). These results suggest that ERMES is dispensable for the PE transport from mitochondria to the ER, and Mmm1 and Mdm34 may have a negative regulatory role in the yet-to-be identified PE transport pathway from mitochondria to the ER.

## Discussion

In the present study, we innovated an *in vitro* assay system for the PS transport from the ER to mitochondria and the PE transport from mitochondria to the ER by using yeast HMFs containing both the ER and mitochondria. By using this assay system, we assessed the energy requirements of the phospholipid transport processes. The roles of energy sources such as ATP in the phospholipid transport were previously studied by using intact cells, permeabilized cells, isolated organelles and liposomes, yet no consensus view was established. Here we found that ATP, GTP and the ∆Ψ are all dispensable for phospholipid transport between the ER and mitochondria (Figs 2 and 3). Therefore, at least in our assay system, the PS transport from the ER to mitochondria and the PE transport from mitochondria to the ER can take place by facilitated passive diffusion without an input of external energy.

Although the role of ERMES in the phospholipid transport was controversial, the present study on the *in vitro* phospholipid transport assays provided direct evidence for the involvement of ERMES components in the PS transport from the ER to mitochondria. In the absence of Mmm1, Mdm12 or Mdm34 the PS transport from the ER to mitochondria was significantly impaired (Figs 4, S4 and S7). Since mitochondria possess multiple membrane contacts with the ER and vacuole, such as EMC and vCLAMP, in addition to ERMES, the loss of ERMES components could be compensated by activation of back-up phospholipid transport routes via such redundant inter-organellar contacts, which would make the phospholipid transport analyses problematic *in vivo.* In contrast, our *in vitro* system appears to overcome such a problem because the HMFs mainly contain the ER and mitochondria, and we could clearly observe the defects in the PS transport upon depletion of Mmm1, Mdm12 or Mdm34. We also confirmed that loss of Vps39, which is a component of vCLAMP did not affect PS transport *in vitro* (Fig. S8). Nevertheless with the present phospholipid transport system, small amounts of PS are still transported from the ER to mitochondria in the absence of Mmm1, Mdm12 or Mdm34 (Figs 4, S4 and S7), suggesting the presence of uncharacterized phospholipid transport route(s) that is independent of ERMES.

In contrast to the PS transport, we found that the PE transport from mitochondria to the ER was not affected with the HMF from *mdm12*∆ cells or was rather enhanced with *mmm1*∆ and *mdm34∆* HMFs (Figs. 4C and S4C (PDME+PC)/PE). This observation raises an intriguing possibility that ERMES differently affects the ER to mitochondria and the mitochondria to ER phospholipid transport processes. It is thus likely that the PE transport is primarily mediated by uncharacterized factors other than ERMES. Of note, the synthesis of PS, which is reflected in the amounts of total radioactive phospholipids, is also suppressed in *mmm1*∆, *mdm12*∆, *mdm34∆* and *mmm1∆mdm12*∆ HMFs (Figs 4, S4 and S7). Immunoblotting reveled that the comparable amounts of Cho1 are present in the HMFs from *mmm1*∆, *mdm12*∆ and *mmm1∆mdm12*∆ cells (Figs 4, S4 and S7). Since the PS transport from the ER to mitochondria became defective in the absence of Mmm1, Mdm12 or Mdm34, a negative feedback regulation may operate for the Cho1 activity for the maintenance of the constant level of PS in the ER membrane. Indeed, we observed that PS accumulates in *mmm1*∆ and *mdm12*∆ HMFs at the steady-state level (Fig. S7D,E). Alternatively, decreased PE levels in the HMFs from *mmm1*∆ and *mdm12*∆ cells as compared with the HMF from wild-type cells (Fig. S7D,E) may suppress the Cho1 activity.

Although our *in vitro* phospholipid transport system allowed us to demonstrate that ERMES plays an important role in PS transport from the ER to mitochondria, there still remain many open questions. For example, whether or not the ERMES complex directly mediates the lipid transfer is still not clear. To directly demonstrate the lipid transfer function of ERMES, we need to examine whether purified ERMES protein or complex functions as a lipid transfer machinery. The residual PS transport route from the ER to mitochondria in the absence of Mmm1, Mdm12 and Mdm34 is unclear, and furthermore the major route for the PE transport from mitochondria to the ER is completely unknown. It is also open how phospholipids flip/flop takes place in the mitochondrial OM and IM. We believe that the present optimal *in vitro* assay system can be used as a powerful tool to answer those questions in future studies.

## Materials and Methods

### Yeast strains, Plasmids, Media and Genetic Methods

FY833 was used as wild-type yeast strains^46^. *cho1∆*, *psd1∆*, *cho2∆*, and *opi3∆* strains were obtained from Open Biosystems. To obtain *mmm1∆mdm12∆* cells, haploid *mmm1∆* and *mdm12∆* cells^20^ were mated and subjected to tetrad dissection. Yeast cells were grown in YPD (1% yeast extract, 2% polypeptone, and 2% glucose), YPLac (1% yeast extract, 2% polypeptone, 3% lactic acid pH 5.6) and SCD (0.67% yeast nitrogen base without amino acids, 0.5% casamino acid, 2% glucose) supplemented with 20 μg/ml each of adenine, uracil, L-tryptophan and L-histidine and 30 μg/ml each of L-leucine and L-lysine.

### Isolation of membrane fractions

Five-hundred μl or 15 ml of overnight yeast preculture was inoculated to 1 L of YPD, SCD or YPLac, respectively and cultivated at 30 °C for 15 h. The typical optical density (OD_600_) after the cultivation was around 1.5. Yeast cells were harvested and incubated in alkaline buffer (0.1 M Tris-HCl, pH 9.5, 10 mM dithiothreitol (DTT)) for 15 min at 30 °C. After washing with spheroplast buffer (20 mM Tris-HCl, pH 7.5, 1.2 M sorbitol), the cells were treated with Zymolyase 20T (5 mg for 1 g yeast wet weight) in spheroplast buffer for 30 min at 30 °C. The resulting spheroplasts were re-suspended in ice-cold breaking buffer (20 mM Tris-HCl, pH 7.5, 0.6 M mannitol, 1 mM EDTA, 1 mM phenylmethylsulfonyl fluoride (PMSF)) and homogenized 20 times with a dounce tissue grinder. After removing unbroken cells and cell debris including nuclear membranes by centrifugation at 2,000× *g* for 5 min, heavy membrane fractions (HMFs) containing the ER and mitochondria were precipitated by centrifugation at 12,000× *g* for 10 min. The HMFs were washed with SEM buffer (250 mM sucrose, 10 mM MOPS-KOH, pH 7.2, 1 mM EDTA), re-suspended in SEM buffer and frozen in liquid nitrogen. The supernatant fraction after the HMFs sedimentation was centrifuged at 25,000× *g* for 30 min and then 40,000× *g* for 30 min to obtain 25 k and 40 k light membrane fractions (LMFs), respectively. Protein concentrations of the membrane fractions were calculated by OD_280_ measurements. Ten μl of the membrane suspension was added to 990 μl of 0.6% SDS and boiled for 5 min. Then, Abs_280_ of the sample was measured. OD_280_ = 0.21 was assumed to be 10 mg/ml proteins.

### *In vitro* phospholipid transport assay between the ER and mitochondria

Assay conditions for the basic *in vitro* phospholipid transport are as follows. Isolated membrane fractions (200 μg protein) in 98 μl of PS transport buffer were pre-incubated at 30 °C for 2 min and then PS synthesis was started by adding 2 μl of 100 μCi/ml [^14^C(U)]-L-serine. The optimized buffer condition is as follows; 300 mM sucrose, 20 mM Tris-HCl, pH 7.5, 40 mM KCl, 2 mM CTP, 1 mM S-adenosylmethionine, 0.1 mM MnCl_2_, 2 mM MgCl_2_. To deplete ATP, 200 μg of HMFs were treated with 1 unit apyrase (New England Biolabs) or 5 units hexokinase (Nacalai tesque) at 30 °C for 10 min in 100 μl of apyrase buffer (300 mM sucrose, 20 mM PIPES-KOH, pH 6.5, 0.1 mM MgCl_2_, 50 mM NaCl) or hexokinase buffer (300 mM sucrose, 20 mM Tris-HCl, pH 7.5, 0.25 mM MgCl_2_, 50 mM NaCl, 10 mM glucose), respectively. The PS synthesis and following transport reactions were stopped by addition of 900 μl of 2:1 chloroform/methanol and vortexing for 15 min, then 200 μl of 0.1M KCl, 0.1 M HCl was added to the samples and further vortexed for 15 min at room temperature. Subsequently after 5 min spin, the organic phase was collected and dried under N_2_ gas. The resulting lipid film was dissolved in chloroform and subjected to the analysis by thin-layer chromatography (TLC) followed by radioimaging. Amounts of PS, PE, PDME and PC were determined using the ImageQuant software (GE Healthcare). TLC plates were purchased from MACHEREY-NAGEL.

### Antibodies

Antibodies against Cds1, Cho1, and Opi3 were raised in rabbits with synthetic peptide MSDNPEMKPHGTSKEIVC (residues 1–17 of Cds1), CHTDTDVIVNEHRDENDG (residues 15–31 of Cho1), or CPFTAMIYANRDKAKKNM (residues 190–206 of Opi3), respectively, which was conjugated to keyhole limpet haemocyanin (KLH), and those against Psd1 and Cho2 with a purified recombinant protein, N-terminally hexahistidine-tagged Psd1 (residues 143–500) or Cho2 (residues 572–869), respectively, which was expressed in *E. coli* cells as inclusion bodies.

### Immunoblotting

For immunoblotting, proteins transferred to PVDF membranes (Immobilon-FL, Millipore) were detected by fluorophores conjugated to secondary antibodies (Cy5 or Alexa Fluor 488 goat anti-rabbit or mouse IgG (H+L) from Thermo Fisher Scientific) and analyzed with a Typhoon imager (GE Healthcare).

## Additional Information

**How to cite this article**: Kojima, R. *et al.* A phospholipid transfer function of ER-mitochondria encounter structure revealed in vitro. *Sci. Rep.* **6**, 30777; doi: 10.1038/srep30777 (2016).

## Supplementary Material

Supplementary Information
