# Supplementary material for: A phospholipid transfer function of ER-mitochondria encounter structure revealed in vitro
Source: Sci Rep. 2016 Jul 29;6:30777. doi: 10.1038/srep30777 (PMC4965753; doi:10.1038/srep30777)
Supplement: Supplementary Information [file srep30777-s1.pdf]

Supplementary Information

A phospholipid transfer function of ER-mitochondria encounter structure revealed in vitro

Rieko Kojima[1], Toshiya Endo[2]* & Yasushi Tamura[1]**

[1]Department of Material and Biological Chemistry, Faculty of Science, Yamagata University, Yamagata, Yamagata 990-8560, Japan.

[2]Faculty of Life Sciences, Kyoto Sangyo University, Kamigamo-motoyama, Kita-ku, Kyoto 603-8555, Japan.

*Correspondence and requests for materials should be addressed to Y.T. (email:tamura@sci.kj.yamagata-u.ac.jp) or T.E. (email:tendo@cc.kyoto-su.ac.jp)

Contents

Supplementary figures 1-8

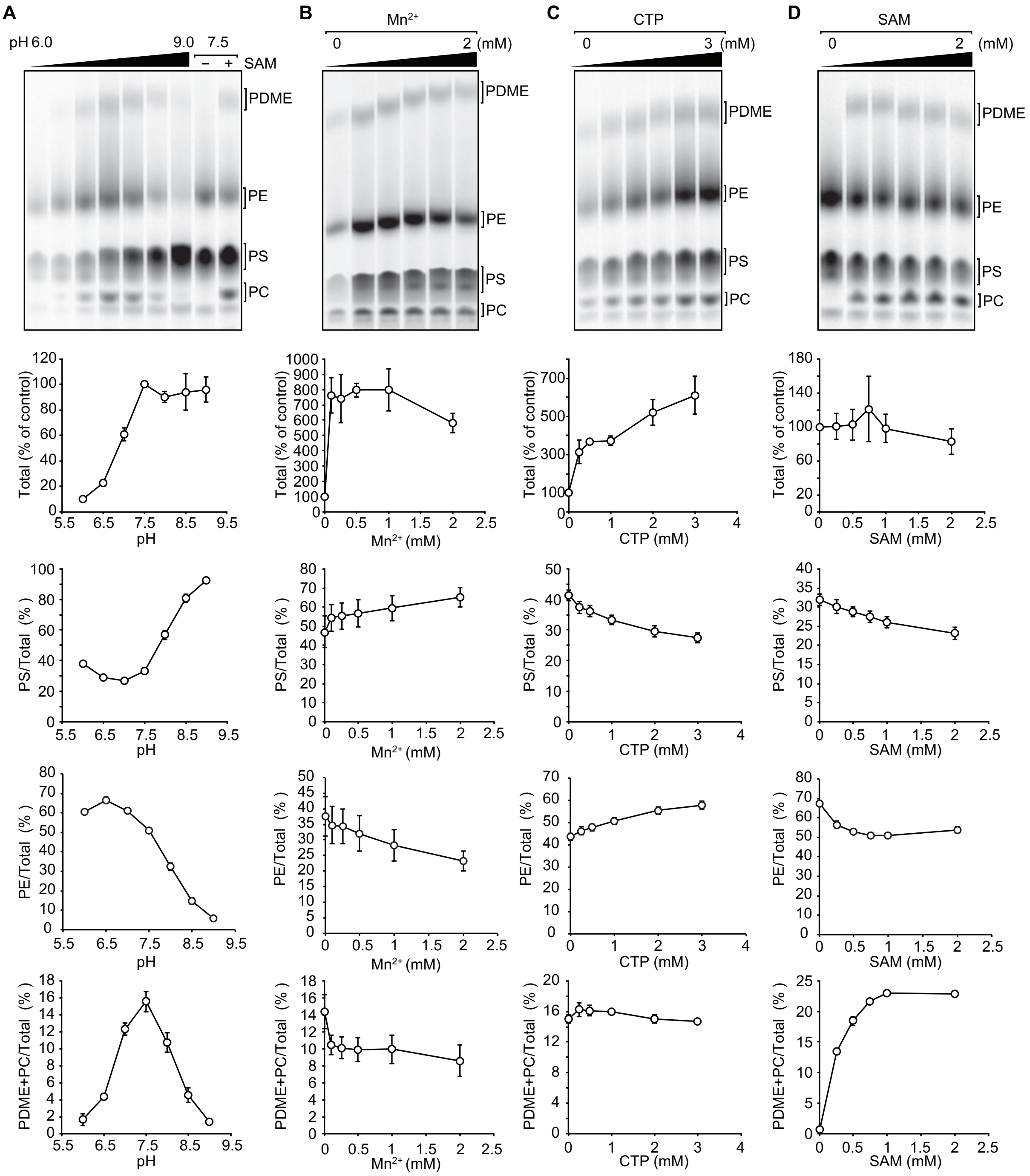

Figure S1. Determination of optimal conditions for *in vitro* PS synthesis and transport. (**A-D**) *In vitro* PS synthesis was performed at different pH (**A**), and in the presence of various concentrations of Mn^{2+} (**B**), CTP (**C**) or SAM (**D**). After 30 min incubation at 30°C, phospholipids were analyzed by TLC and radioimaging.

When pH was increased, the PS synthesis (Total) increased to reach plateau at pH 7.5 (A, second upper panel). While the PS synthesis remained maximal at pH over 7.5, the ratio of radioactive PS to total phospholipids increased as pH increased (A, central panel),

suggesting that PS transport to the IM and/or conversion of PS to PE became less efficient as pH increased. Amounts of radioactive PE, which depend on the PS transport as well as PS conversion to PE, were maximal at pH 6.5 and then decreased with pH (A, second lower panel). Amounts of radioactive PDME and PC, which depend on the transport of PE from mitochondria to the ER, were maximal at pH 7.5 (A, lowermost panel). On the basis of these observations, we adopted pH 7.5 as the optimal pH to monitor PS and PE transport processes *in vitro*.

We found that 0.1 mM Mn^{2+} is sufficient to observe efficient PS synthesis (B). We also tested the requirement of CTP and SAM for PS synthesis and subsequent PS and PE transport processes *in vitro* (C, D). PS synthesis increased with CTP concentrations (Total) while the presence of CTP only slightly accelerated the conversion of PS to PE or PC. We thus set the CTP concentration to 2 mM for further analyses. Since SAM provides methyl groups for production of PDME and PC (Fig 1A), the presence of SAM is prerequisite for i*n vitro* conversion of PE to PDME and PC, which is reflected in the relative amounts of radioactive PDME and PC to the synthesized total phospholipids (lowermost panel), but not for PS synthesis on its own (uppermost panel). We thus set the SAM concentration to 1 mM for further analyses.

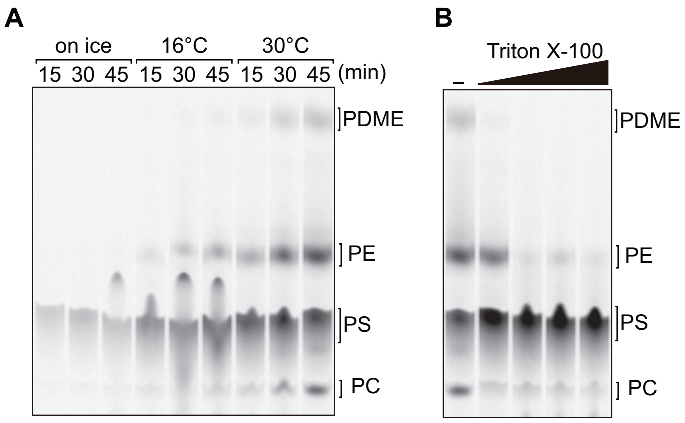

Figure S2. Physiological temperature and membrane intactness are required for *in vitro* PS synthesis and transport. (**A**) 12k pellet fractions as prepared in Fig. 1D were incubated with [^{14}C]-serine on ice, at 16°C or 30°C for the indicated times. Total phospholipids were extracted and analyzed by TLC and radioimaging. (**B**) 12k pellet fractions as prepared in Fig. 1D, were incubated with [^{14}C]-serine in the absence or presence of 0.01, 0.025, 0.05 or 0.1% Triton X-100 at 30°C for 45 min. Total phospholipids were extracted and analyzed by TLC and radioimaging.

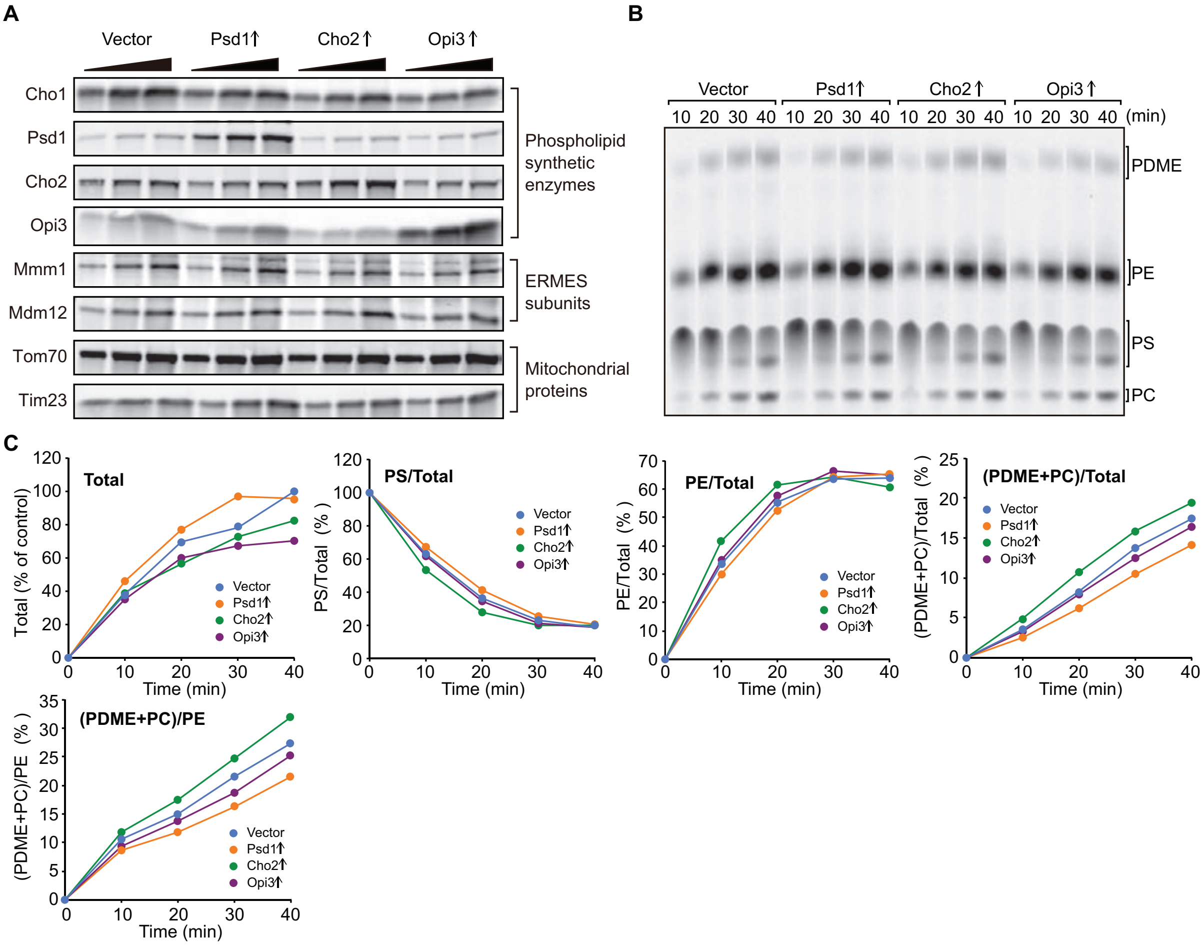

Figure S3. Increased levels of phospholipid synthetic enzymes do not affect *in vitro* PS synthesis and transport. (**A**) Proteins in the heavy membrane fractions isolated from wild-type cells with an empty *2μ* vector pRS424 (Vector) or pRS424 plasmid harboring the *PSD1* (Psd1↑), *CHO2* (Cho2↑) or *OPI3* (Opi3↑) gene were analyzed by SDS-PAGE followed by immunoblotting using the indicated antibodies. (**B**) *In vitro* PS synthesis and transport assays were performed using the heavy membrane fractions in (A). (**C**) The amount of total phospholipids synthesized in vector control after 40 min incubation was set to 100% (Total). Amounts of PS, PE and PDME+PC relative to total phospholipids were calculated and plotted.

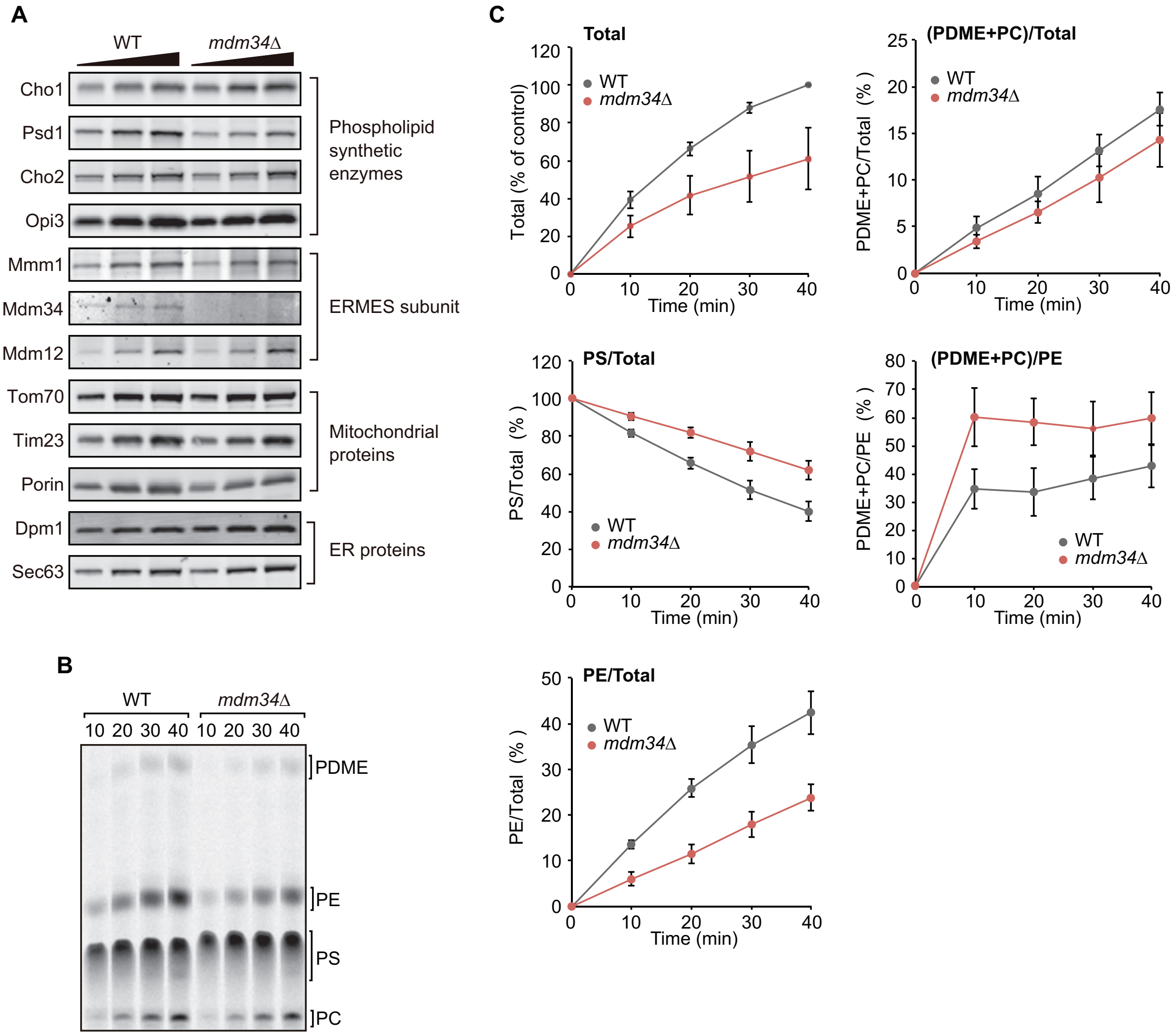

Figure S4. Mdm34 facilitates phospholipid transport between the ER and mitochondria. (**A**) Proteins in the heavy membrane fractions isolated from wild-type and *mdm34*Δ cells expressing Vps13-D716H were analyzed by SDS-PAGE followed by immunoblotting using the indicated antibodies. (**B**) *In vitro* PS transport assays were performed using the heavy membrane fractions isolated from wild-type and *mdm34*Δ cells expressing Vps13-D716H. (**C**) Amounts of PS, PE and PDME+PC relative to total phospholipids were calculated and plotted. Values are mean ± SEM (n=4). The amount of total phospholipids synthesized with wild-type cells after 40 min incubation was set to 100% (Total).

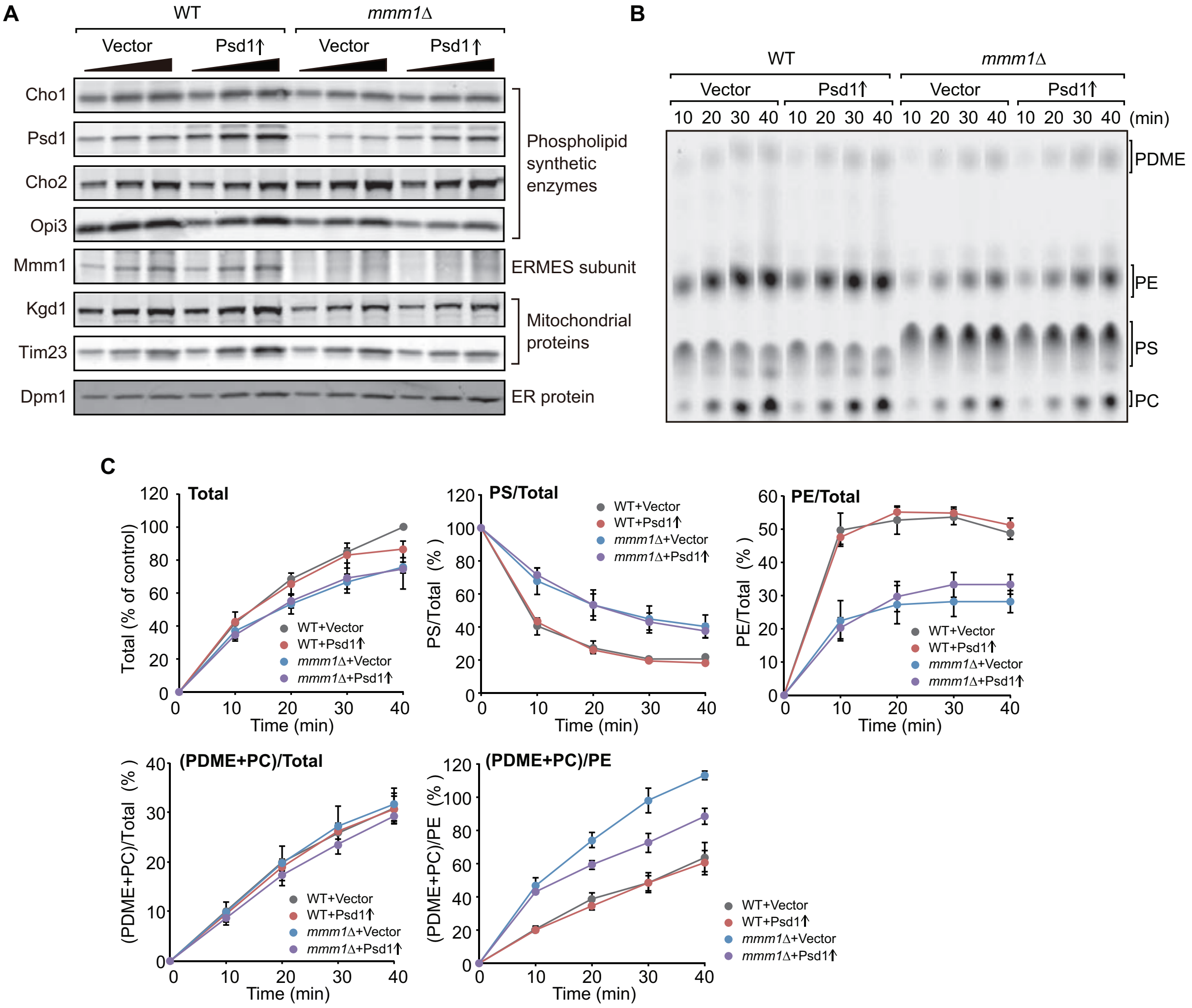

Figure S5. Psd1 overexpression does not affect the PS transport rate in the absence of Mmm1. (**A**) Proteins in the heavy membrane fractions isolated from wild-type or *mmm1*Δ cells with an empty *2μ* vector pRS426 (Vector) or pRS426 plasmid harboring the *PSD1* (Psd1↑) gene were analyzed by SDS-PAGE followed by immunoblotting using the indicated antibodies. (**B**) *In vitro* PS synthesis and transport assays were performed using the heavy membrane fractions in (A). (**C**) Amounts of PS, PE and PDME+PC relative to total phospholipids were calculated and plotted. The amount of total phospholipids synthesized in vector control after 40 min incubation was set to 100% (Total). Values are mean ± SEM (*n*=3).

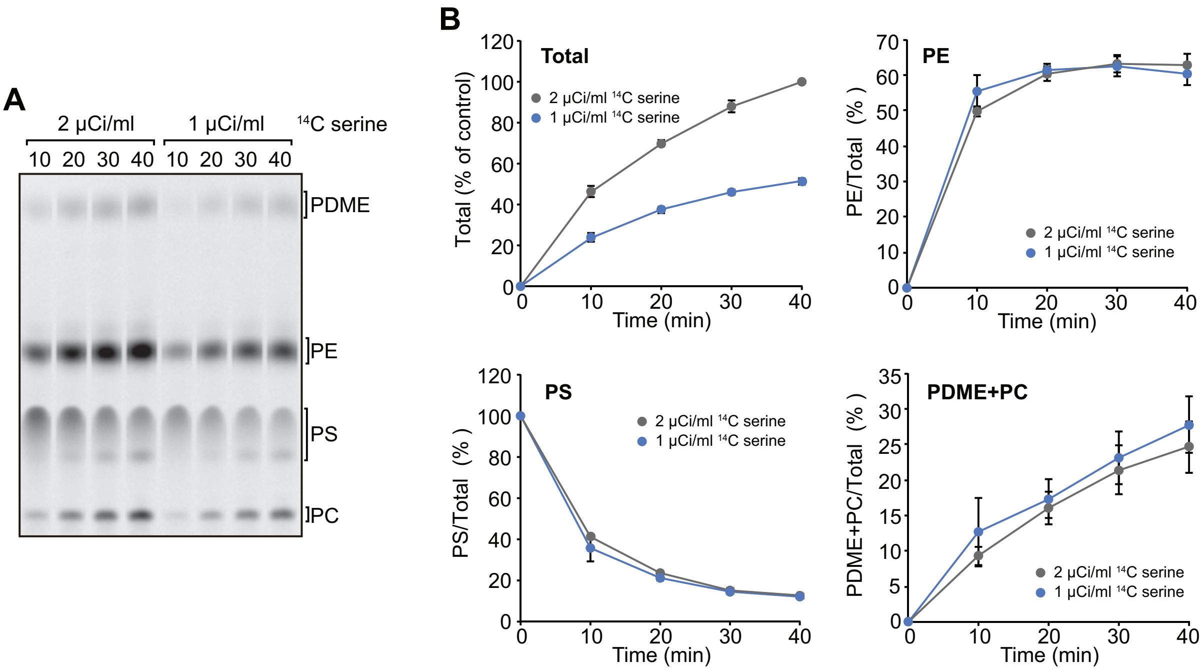

Figure S6. Decreased level of PS synthesis does not affect the rate of the PS transport. (**A**) PS was synthesized *in vitro* using 2 μCi/ml or 1 μCi/ml [^{14}C]-serine and analyzed by TLC after the indicated incubation periods. (**B**) The amount of total phospholipids synthesized with wild-type membranes after 40 min incubation was set to 100% (Total). Amounts of PS, PE and PDME+PC relative to total phospholipids were calculated and plotted. Values are mean ± SEM (*n*=3).

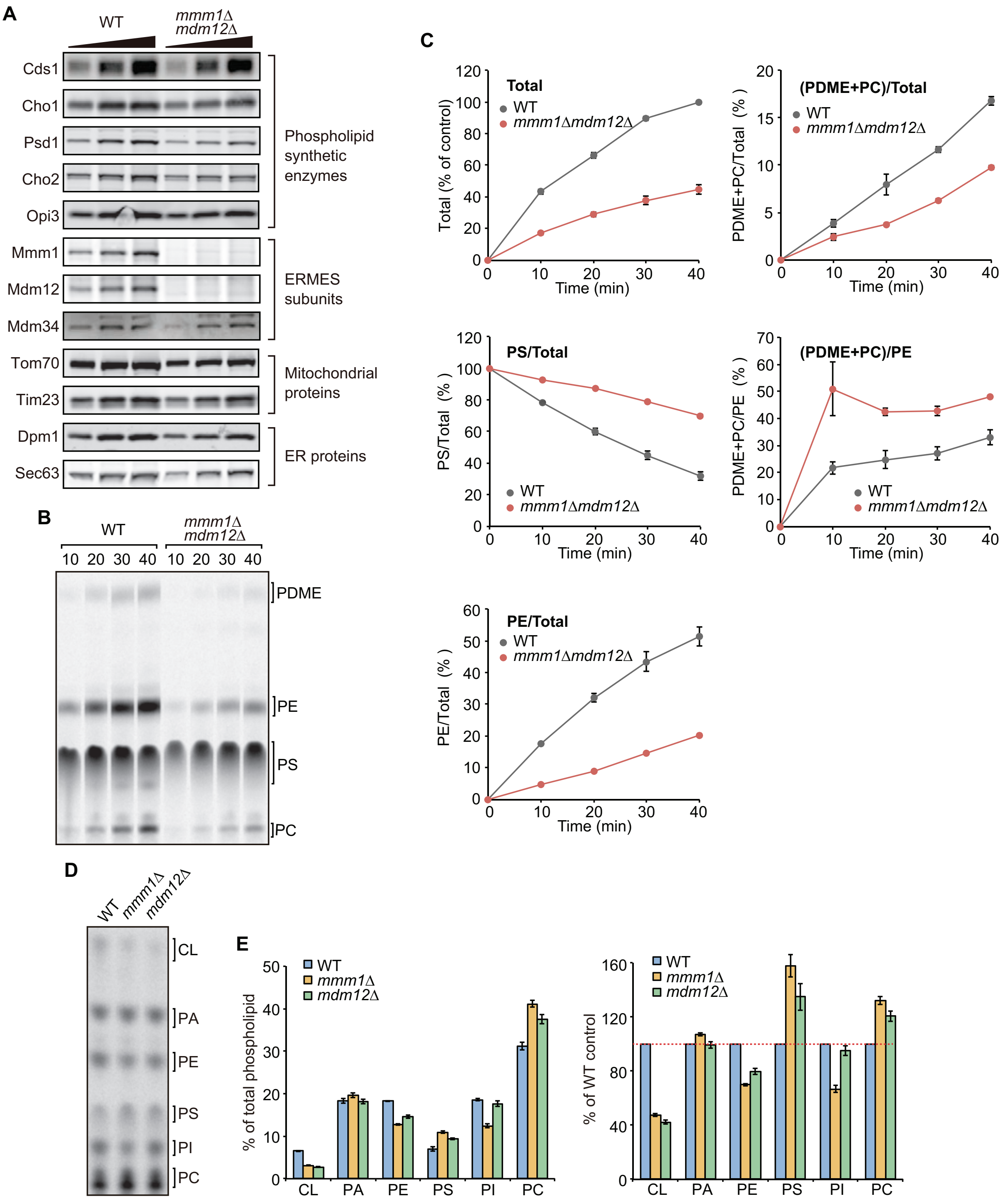

Figure S7. *mmm1Δmdm12Δ* cells decelerated the PS transport and accelerated the PE transport. (**A**) Proteins in the heavy membrane fractions isolated from wild-type and *mmm1Δmdm12Δ* cells expressing Vps13-D716H were analyzed by SDS-PAGE followed by immunoblotting using the indicated antibodies. (**B**) *In vitro* PS synthesis and transport assays were performed using the heavy membrane fractions in (A). (**C**) Amounts of PS, PE and PDME+PC relative to total phospholipids were determined and

plotted. The amount of total phospholipids synthesized with wild-type membranes after 40 min incubation was set to 100% (Total). Values are mean ± SEM (*n*=3). (**D**) Wild-type, *mmm1*Δ, and *mdm12*Δ cells expressing Vps13-D716H were grown in 2 ml SCD media in the presence of 2 μCi/ml ^{32}P to stationary phase. Phosphoipids were extracted from the heavy membrane fractions containing mitochondria and the ER membranes and analyzed by TLC followed by radioimaging. (**E**) Amounts of each phospholipid in (D) were measured and relative amounts to total phospholipids were determined (left panel). Ratios of each phospholipid in *mmm1*Δ and *mdm12*Δ cells to that in wild-type cells were set to 100% (right panel). Values are mean ± SEM (*n*=4).

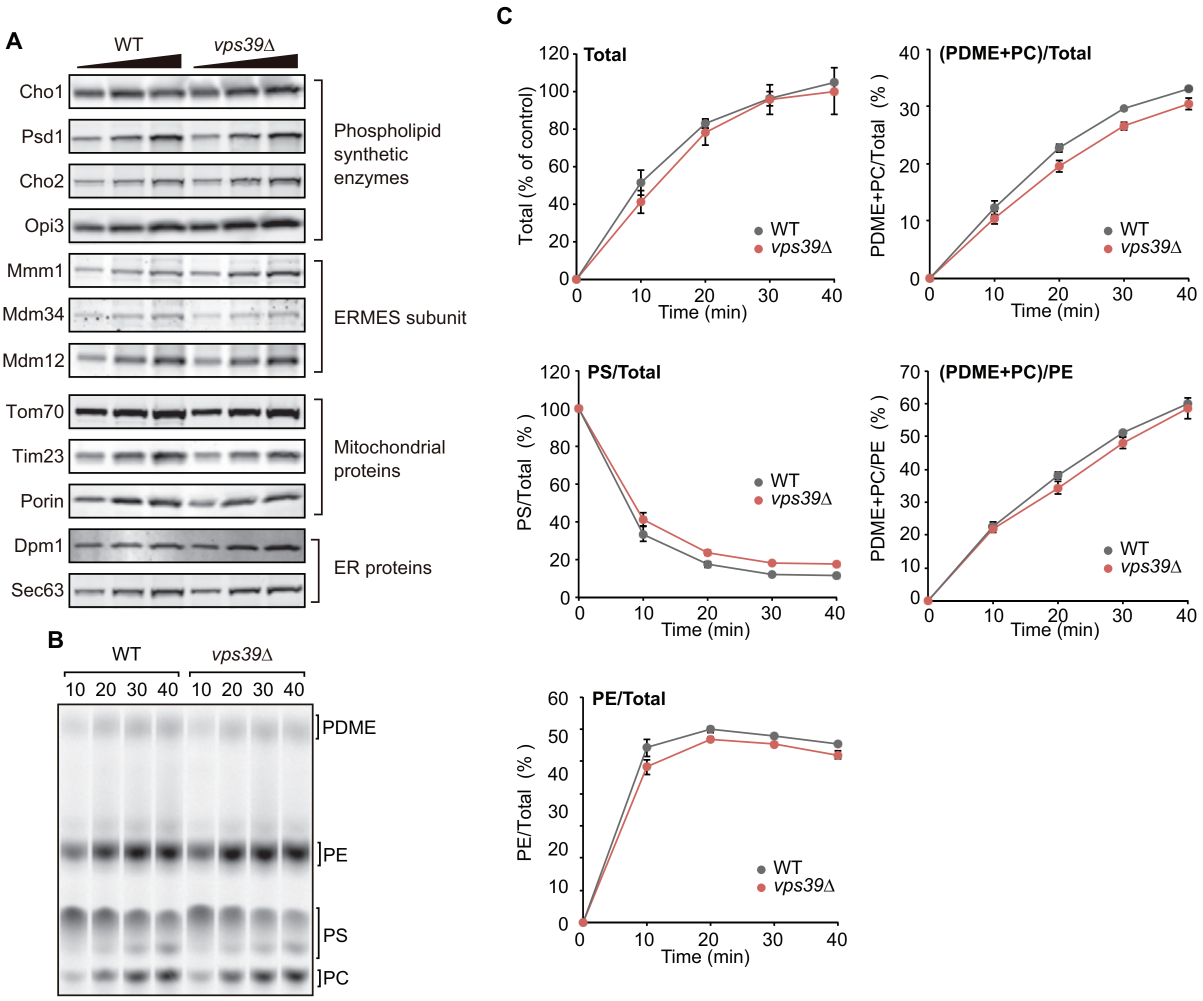

Figure S8. Loss of Vps39 does not affect PS transport *in vitro*. (**A**) Proteins in the heavy membrane fractions isolated from wild-type and *vps39*Δ cells were analyzed by SDS-PAGE followed by immunoblotting using the indicated antibodies. (**B**) *In vitro* PS transport assays were performed using the heavy membrane fractions isolated from wild-type and *vps39*Δ cells. (**C**) Amounts of PS, PE and PDME+PC relative to total phospholipids were calculated and plotted. Values are mean ± SEM (n=4). The amount of total phospholipids synthesized with wild-type cells after 40 min incubation was set to 100% (Total).
